# Supplementary material for: Assessing inhibitors of mutant isocitrate dehydrogenase using a suite of pre-clinical discovery assays
Source: Sci Rep. 2017 Oct 6;7:12758. doi: 10.1038/s41598-017-12630-x (PMC5630632; doi:10.1038/s41598-017-12630-x)

Assessing inhibitors of mutant isocitrate dehydrogenase using a suite of pre-clinical discovery assays.

Daniel J. Urban, Natalia J. Martinez, Mindy I. Davis, Kyle R. Brimacombe, Dorian M. Cheff, Tobie D. Lee, Mark J. Henderson, Steven A. Titus, Rajan Pragani, Jason M. Rohde, Li Liu, Yuhong Fang, Surendra Karavadhi, Pranav Shah, Olivia W. Lee, Amy Wang, Andrew McIver, Hongchao Zheng, Xiaodong Wang, Xin Xu, Ajit Jadhav, Anton Simeonov, Min Shen, Matthew B. Boxer, Matthew D. Hall

SUPPLEMENTARY INFORMATION

Supplemental Methods

Compound Synthesis -

Sanofi 1 compound (1) was synthesized as follows:

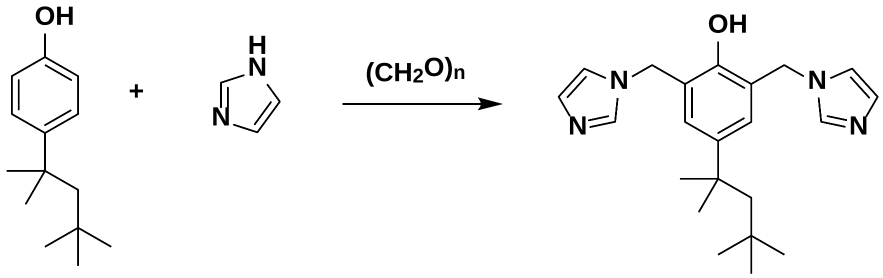

A solution of 4-(2,4,4-trimethylpentan-2-yl)phenol (2.0 g, 9.69 mmol), paraformaldehyde (1.164 g, 38.8 mmol), and 1H-imidazole (1.980 g, 29.1 mmol) in EtOH (10 mL) was heated at reflux for 1 h. Then the solvents were removed under reduced pressure to give an oil that was then heated at ~130 °C for 6 h. The reaction mixture was dissolved in methanol and added to 40 ml of 20% brine solution. The resulting solution was heated to reflux and the pH was adjusted to 3 with 2N HCl, and then the reaction mixture was cooled to room temperature. The residue was taken up in DMSO and subsequently purified by reverse phase chromatography to give 1.74 g (49% yield) of 2,6-bis((1H-imidazol-1-yl)methyl)-4-(2,4,4-trimethylpentan-2-yl)phenol as a white solid. 1H NMR (400 MHz, methanol-d4) δ 7.66 (s, 2H), 7.06 (s, 4H), 6.94 (s, 2H), 5.22 (s, 4H), 4.83 (s, 1H), 1.63 (s, 2H), 1.24 (s, 6H), 0.60 (s, 9H); LCMS: m/z (M+H) = 367.0.

Novartis 530 was synthesized as follows:

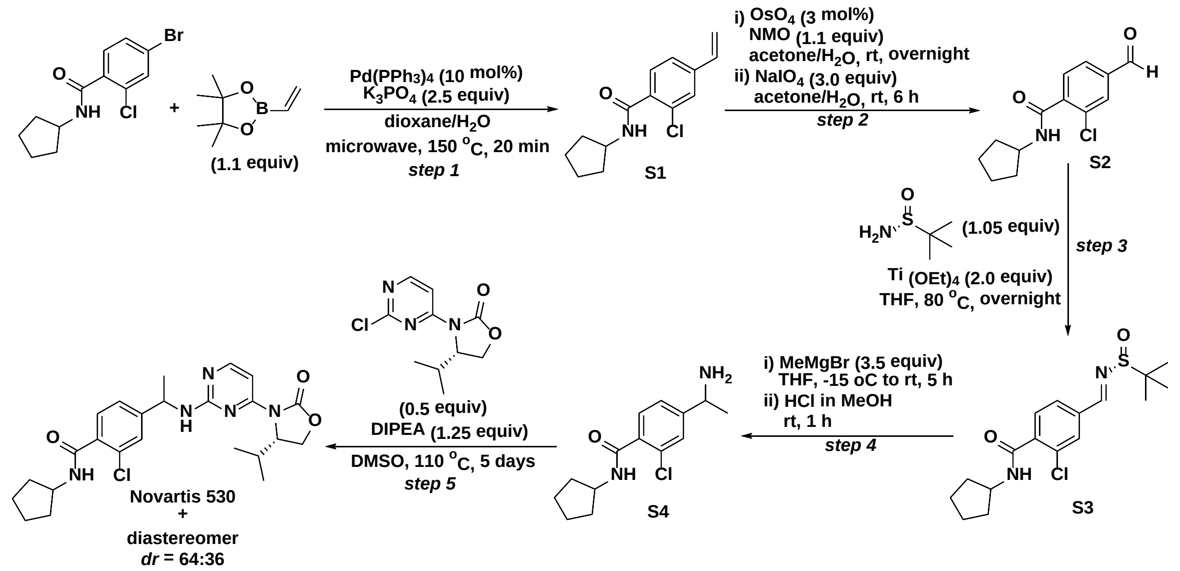

Step 1: A mixture of 4-bromo-2-chloro-N-cyclopentylbenzamide (1.2 g, 3.97 mmol), 4,4,5,5-tetramethyl-2-vinyl-1,3,2-dioxaborolane (0.74 mL, 4.36 mmol), palladium tetrakis(triphenylphosphine) (459 mg, 0.4 mmol) and potassium phosphate (2.1 g, 9.93 mmol) in dioxane/H2O (16 mL/4 mL) was heated at 150 °C under microwave radiation for 20 min. Subsequently, the solvents were removed under reduced pressure. The residue was purified by column chromatography with a pre-packed, disposable silica gel column to afford the desired product S1 (708 mg, 71% yield). 1H NMR (400 MHz, CDCl3) δ 7.66 (d, J = 8.0 Hz, 1H), 7.40 (d, J = 1.6 Hz, 1H), 7.33 (dd, J = 8.0, 1.6 Hz, 1H), 6.65 (dd, J = 17.6, 10.9 Hz, 1H), 6.20 (br s, 1H), 5.80 (d, J = 17.6 Hz, 1H), 5.38 (d, J = 10.9 Hz, 1H), 4.47-4.37 (m, 1H), 2.13-2.03 (m, 2H), 1.80-1.60 (m, 4H), 1.55-1.50 (m, 2H); MS (ESI) for [M+H]+ (C14H17ClNO): calcd. m/z 250.10; found m/z 250.10; LC-MS: T = 5.25 min, >95% purity.

Step 2: To a solution of S1 (708 mg, 2.83 mmol) in acetone (14 mL) at room temperature was added a solution of N-methylmorpholine-N-oxide (421 mg, 3.11

mmol) in H_2O (14 mL) and osmium tetroxide (21.6 mg, 0.089 mmol). The resulting mixture was stirred at room temperature overnight. The volatiles were removed under reduced pressure. The residue was neutralized with a 2 M aqueous solution of H_2SO_4. The resulting mixture was extracted with EtOAc (2 × 20 mL), dried (Na_2SO_4), filtered, and concentrated. The residue was dissolved in a mixture of acetone (14 mL) and H_2O (14 mL) and to it was added sodium periodate (1.8 g, 8.99 mmol). The resulting mixture was stirred at room temperature for 6 h. Then the solvents were removed under reduced pressure. The residue was purified by column chromatography with a pre-packed, disposable silica gel column to afford the desired product S2 (395 mg, 55% yield). 1H NMR (400 MHz, $CDCl_3$) δ 10.00 (s, 1H), 7.90-7.87 (m, 1H), 7.83-7.75 (m, 2H), 6.08 (br s, 1H), 4.49-4.39 (m, 1H), 2.15-2.05 (m, 2H), 1.80-1.50 (m, 6H); MS (ESI) for [M+H]+ ($C_{13}H_{15}ClNO_2$): calcd. m/z 252.08; found m/z 252.08; LC-MS: T = 4.86 min, >95% purity.

Step 3: To a solution of S2 (395 mg, 1.57 mmol) in THF (2.6 mL) was added (R)-2-methylpropane-2-sulfinamide (200 mg, 1.65 mmol) and $Ti(OEt)_4$ (0.66 mL, 3.14 mmol) at room temperature. The resulting reaction mixture was heated at 80 °C overnight, then was extracted between H_2O (30 mL) and EtOAc (3 × 30 mL). The combined organic layers were washed with brine (20 mL), dried (Na_2SO_4), filtered, and concentrated. The residue was purified by column chromatography with a pre-packed, disposable silica gel column to afford the desired product S3 as a white solid (466 mg, 84% yield). MS (ESI) for [M+H]+ ($C_{17}H_{24}ClN_2O_2$): calcd. m/z 355.12; found m/z 355.12; LC-MS: T = 5.44 min, >95% purity.

Step 4: To a solution of S3 (444 mg, 1.25 mmol) in THF (12.5 mL) was added methylmagnesium bromide solution (3.0 M in Et2O, 1.46 mL, 4.38 mmol) at –15 °C. The reaction mixture was allowed to warm to room temperature and stirred at room temperature for 5 h. A saturated aqueous NH4Cl solution (50 mL) was added to quench the reaction, and the reaction mixture was extracted with EtOAc (3 × 50 mL). The combined organic layers were washed with brine (20 mL), dried (Na2SO4), filtered, and concentrated. The residue was treated with a solution of HCl (3 M in methanol, 3.0 mL) and methanol (12 mL). The resulting solution was stirred at room temperature for 1 h. Upon evaporation of volatiles, the residue was extracted between an aqueous NaOH solution (6 N, 15 mL) and CH2Cl2 (3 × 30 mL). The combined organic layers were washed with brine (20 mL), dried (Na2SO4), filtered, and concentrated to provide the crude S4 as a yellow oil. The crude product S4 was used in the next step without any further purification.

Step 5: A solution of (S)-3-(2-chloropyrimidin-4-yl)-4-isopropyloxazolidin-2-one (174 mg, 0.72 mmol), crude S4 (1.25 mmol) and N,N-diisopropylethylamine (0.32 mL, 1.8 mmol) in DMSO (2.8 mL) was heated at 110 °C for 5 d. The reaction mixture was extracted between H2O (3 × 30 mL) and CH2Cl2 (30 mL). The organic layer was washed with brine (20 mL), dried (Na2SO4), filtered, and concentrated. The residue was purified by column chromatography with a pre-packed, disposable silica gel column to afford a mixture of the desired product, Novartis 530, along with the benzylic methyl diastereomer of Novartis 530 as a light yellow solid (223 mg, 66% yield, dr = 64:36). 1H NMR (400 MHz, CDCl3) δ 8.20-8.10 (m, 1H), 7.64-7.60 (m, 1H), 7.48-7.44 (m, 1H), 7.34-7.22 (m, 2H), 6.20-6.00 (m, 1H), 5.60-5.30 (m, 1H), 5.05-4.80

(m, 1H), 4.60-4.45 (m, 1H), 4.40 -4.20 (m, 3H), 2.70-2.50 (m, 1H), 2.10-2.05 (m, 2H), 1.80-1.45 (m, 8H), 1.00-0.90 (m, 1H), 0.90-0.80 (m, 3H), 0.77-0.50 (m, 3H) ; MS (ESI) for [M+H]+ (C24H31ClN5O3): calcd. m/z 472.21; found m/z 472.21; LC-MS: Tmajor = 5.55 min, Tminor = 5.62 min.

IDH1 and IDH2 biochemical assays:

Wild Type IDH1 and IDH2 enzyme assay

WT IDH1 protocol was performed as previously described (2,3). WT IDH1 (3 µL) in assay buffer (20 mM Tris pH 7.5, 10 mM MgCl2, 150 mM NaCl, and 0.05% protease-free BSA) was added to the black solid bottom 1,536-well assay plate using a BiorapTR flying reagent dispenser (BiorapTR; Beckman Coulter). A pintool (Kalypsys) was used to transfer 23 nL of compound solution (library and control) to the 1,536-well assay plates, and plates were spun down at 1,000 rpm for 1 min. After 30 min of incubation at room temperature, 1 µL of substrate buffer was added to initiate the reaction at final concentrations of 0.045 µg/mL enzyme, 2 mM BME, 240 µM isocitrate, 180 uM NADP+, 60 µg/mL diaphorase, and 37.5 µM resazurin. The plate was rapidly transferred to a ViewLux (PerkinElmer) and the fluorescence product resorufin was measured (excitation = 525 nm, emission = 598 nm) in kinetic mode. For individual experiments, the timing of reads varied depending on the number of plates. In initial optimization experiments, the plates were read continuously from t = 0 to t = 15 min.

WT IDH2 (3 µL) in assay buffer (100 mM Tris pH 7.5, 1.3 mM MnCl2, 0.05 % protease-free BSA, and 10 % glycerol) was added to the black solid bottom 1,536-well assay plate using a BiorapTR flying reagent dispenser (BiorapTR; Beckman

Coulter). A pintool (Kalypsys) was used to transfer 23 nL of compound solution (library and control) to the 1,536-well assay plates. After 30 min of incubation at room temperature, 1 µL of substrate buffer was added to initiate the reaction at final concentrations of 0.0225 µg/mL enzyme, 2 mM BME, 5 µM isocitrate, 30 µM NADP+, 12 µg/mL diaphorase, and 4 µM resazurin. The plate was rapidly transferred to a ViewLux (PerkinElmer) and the fluorescence product resorufin was measured (excitation = 525 nm, emission = 598 nm) in kinetic mode. For individual experiments, the timing of reads varied depending on the number of plates. In initial optimization experiments, the plates were read continuously from t = 0 to t = 5 min.

mIDH1-R132H and IDH-R132C enzyme assay

mIDH1 protocol was performed as previously described (2). Briefly, 3µL of mutant enzyme (R132H (Agios), R132C, (Beryllium)) in assay buffer (R132H: 150 mM NaCl, 20 mM Tris-HCl pH 7.5, 10 mM $MgCl_2$, and 0.05 % protease-free BSA, R132C: 50 mM K_2HPO_4 pH 6.5, 5 mM $MgCl_2$, 0.03 % protease-free BSA, and 10 % glycerol) was added to the black solid bottom 1,536-well assay plate using a BiorapTR. A pintool (Kalypsys) was used to transfer 23 nL of compound solution (library and control) to the 1,536-well assay plates. After 30 min incubation at room temperature, 3 µL of buffer containing NADPH and α-KG was added to initiate the reaction. The final concentrations in the 6 µL reaction volume for R132H contained 0.25 µg/mL enzyme, 2 mM BME, 8 µM NADPH and 1 mM α-KG. The mIDH1 R132C reaction contained 0.5 µg/mL enzyme, 6 µM NADPH and 300 µM α-KG. After a 50 min incubation period for R132H, or a 40 min incubation period for mIDH1 R132C, 3 µL of detection buffer was added to a total volume of 9 µL. Detection buffer contained

60 μg/mL diaphorase and 36 μM resazurin for R132H, or 30 μg/mL diaphorase (Sigma) and 30 μM resazurin for R132C. The plate was transferred to an Envision (PerkinElmer) after 5 min and the fluorescence product resorufin was measured (excitation = 540 nm, emission = 590 nm)

mIDH2-R170Q enzyme assay

Reaction Biology profiled all compounds against mIDH2-R172Q using a diaphorase / resazurin coupled reaction.

Cell culture:

U87 (R132H) and HT1080 were obtained from Agios, THP1 (WT and R132H) were obtained from Ravindra Majeti (Stanford University), SNU1079 and RBE from Nabeel M. Bardeesy (Harvard University), and JJ012 were obtained from Karina Galoian (University of Miami). Short tandem repeat analysis was performed on each cell line using WiCell Research Institute and Genetic Resources Core Facility at the University Johns Hopkins (Supplemental Table 2). HT1080, U87-R132H, and JJ012 cells were cultured in DMEM (Thermo Scientific) supplemented with 10 % FBS, 100 units/mL Penicillin, 100 ug/mL Streptomycin (Thermo Scientific). SNU1079, RBE and THP1-R132H and -WT cells were grown in RPMI 1640 with 2 mM glutamine (Thermo Scientific), supplemented with 10 % FBS and 100 units/mL Penicillin, 100 μg/mL Streptomycin (Thermo Scientific). All cell lines were routinely tested and were negative for mycoplasma contamination (Alert mycoplasma detection kit - Lonza)

2-HG production: Cells grown in monolayer (2D)

HT1080, U87 (R132H), RBE, SNU1079 and JJ012 cells were seeded into clear, TC-treated 96-well plates (Corning) at a density of 8000 cells/well/100 µL. Plates were sealed with gas permeable membranes (Research Products International Corp.) and incubated at 37°C, 5% CO_2, 90% RH for 24 hr. Growth media was subsequently aspirated and replaced with 100 µL of a solution of growth media containing either vehicle DMSO or indicated mIDH1 inhibitor. Each compound was tested as an 8-point dilution series ranging from 5 µM to 2.3 nM. After 48 hr treatment, 75 µL of the exhausted media was collected and snap frozen on dry ice. Samples were analyzed by LC-MS for 2-HG concentrations using Rapidfire / Mass Spectrometry (Quintara) (4). Remaining cells were assessed for viability by adding 100 µL of fresh media and 50 µL of CellTiter-Glo reagent (Promega) as per manufacturer instructions. Luminescence was read using a ViewLux High-throughput CCD imager (PerkinElmer) equipped with clear filters.

2-HG production: Cells grown in suspension

THP1 cells were induced with 2 µg/mL doxycycline for 7 days. Cells were subsequently seeded into clear, TC-treated, 96-well V-bottom plates (Corning) at a density of 20,000 cells/well/100 µL. Plates were sealed with gas permeable membranes and incubated at 37°C, 5% CO_2, 90% RH. After 24 hr plates were centrifuged at 161 rcf for 5 min to pellet the cells. Media was manually removed and replaced with 200 µL of fresh media containing doxycycline and the compounds as indicated above. Cells were immediately resuspended and incubated for 48 hr. Plates were centrifuged at 161 rcf for 5 min and 75 µL of the exhausted media was collected and snap frozen on dry ice. Samples were analyzed by LC-MS for 2-HG

concentrations using Rapidfire / Mass Spectrometry (Quintara) (4). Cell viability was measured using CellTiter-Glo assay as described above.

2-HG production: Cells grown as spheroids (3D)

HT1080, U87 (R132H), RBE, and JJ012 cells were seeded into ultra-low attachment 96-well plates (Corning) at a density of 3,000 (HT1080, U87 and JJ012) or 10,000 (RBE) cells/well/150 µL to form spheroids. Of note, we were not able to obtain spheroids using SNU1079 cells. Plates were sealed with gas permeable membranes, centrifuged at 40 rcf for 1 min, and incubated at 37°C, 5% CO_2, 90% RH for 96 (HT1080, U87 and JJ012) or 48 (RBE) hr. Cells were washed (by removing 100 µL of exhausted media and replacing it with 100 µL of growth media, twice) and treated with 50 µL of a solution of media containing the indicated compounds. Each compound was tested as an 8-point dilution series ranging from 5 µM to 2.3 nM. After 96 hr of treatment, 100 µL of the exhausted media was collected and snap frozen on dry ice. Samples were analyzed by LC-MS for 2-HG concentrations using Rapidfire /Mass Spectrometry (Quintara) (4). Plates were first imaged on the Celigo Imaging Cytometer (Nexcelom Bioscience) to quantify area of each spheroid. Cell viability was subsequently measured using the CellTiter-Glo assay with some modifications: 50 µL of a solution of CellTiter-Glo reagent containing a final concentration of 0.025 % Trypsin (Thermo Scientific) was added to each well and plates were shaken at 500 rpm for 30 min before reading.

Diaphorase coupled D-2-Hydroxyglutarate Dehydrogenase Assay:

The diaphorase coupled D-2-hydroxyglutarate dehydrogenase assay was a modification of the report by Jorg Balss and colleagues (5). HT1080 cells were dispensed into 384-well TC-treated cell culture plates at a density of 4,000 cells/well in 20 µL DMEM (without phenol red), supplemented with 1 % fetal bovine serum and 100 units/mL Penicillin, 100 µg/mL Streptomycin using a Multidrop Combi (Thermo Fisher Scientific). A 384 pintool (Kalypsys) was used to transfer 96 nL of compound solution to the 384-well assay plates. After 48 hr incubation at 37°C, the plates were shaken at 500 rpm for 5 min before 10 µL of media was transferred to new 384-well white solid bottom assay plates using a CyBi-well liquid handling system (CyBio). Collected media was neutralized by transferring 1 µL HCl (final concentration 60 mM) to the plates followed by a 10 min incubation and 1 µL Tris-base (final concentration 60 mM) using a CyBi-well (CyBio). 36 µL of detection solution, containing 100 mM HEPES, pH 8.0, 100 µM NAD^+, 1 µg/mL active recombinant D2HGDH (BioVision, cat: P1001), 5 µM resazurin, 0.03 mg/mL diaphorase (Sigma, cat: D5540), was then added to the 12 µL of neutralized media. After 30 min incubation at room temperature, the plates were transferred to a Viewlux HTS Microplate Imager (PerkinElmer) and the fluorescence product resorufin was measured for 2 seconds (excitation=525, emission=598/25).

Cellular thermal shift assays:

U87 mIDH1-R132H thermal melt

The cellular thermal shift assay and the isothermal dose response was run as previously described (6). Briefly, U87-R132H cells were dispensed into thin-walled

PCR tubes at a density of ~50,000 cells / tube in 100 μL of DMEM (without phenol red), supplemented with 4.5 g/L of glucose and 100 units/mL Penicillin, 100 μg/mL Streptomycin. Cells were subjected to 3 min of heat in 96-well thermal cycler (Veriti – Applied Biosystems) at temperatures of 37, 43, 46, 49, 52, 55, 58, 61, 64, 67, 70, and 73°C. After heating, cells were left to cool for 3 min and then snap frozen in a CoolSafe Chamber (USA Scientific) surrounded by dry ice. Cells were subjected to 3 rapid freeze thaws with 15 seconds of vortexing after each thaw. After the first thaw, 1 μL of protease inhibitor cocktail (Sigma – P8340) was added to the cell lysate. Cell lysates were centrifuged at 20,000 x g for 20 min. Samples were prepared for gel-electrophoresis and western blot analysis by transferring 50 μL of supernatant into 17 μL of 4X SDS Sample Buffer (200 mM Tris-HCl pH 6.8, 8 % SDS, 0.4 % Bromophenol Blue, 40 % glycerol, and 400 mM β-mercaptoethanol) and heated for 10 min at 70°C. Samples were separated on a NuPAGE™ Novex™ 4-12% Bis-Tris gel and transferred to PVDF using an iBlot 2 Dry Blotting System. Membranes were blocked for 1 hr with 5% milk before being incubated at 4°C overnight with 1:500 of mouse monoclonal anti-IDH1-R132H (Millipore – MABC171) and 1:2500 of rabbit polyclonal anti-GAPDH (Abcam – ab9485) in 5% milk. Blots were washed 3 x and incubated with 1:1000 horse anti-mouse HRP linked IgG (Cell Signaling - 7076) for 1 hr at room temperature. Blots were imaged after washing 3 x and incubation with SuperSignal™ West Dura Extended Duration Substrate (Thermofisher -34076). Densitometry analysis was performed using ImageQuant TL (GE Healthcare) and melting curve analysis was performed using Prism (Graphpad Software).

Isothermal Dose Response of mIDH1 inhibitors

U87-R132H cells were dispensed into thin-walled PCR tubes as describe above with the addition of 1 μL of mDH1 inhibitors in DMSO for a final 7-point concentration range of 100 μM – 0.024 μM. Samples were incubated at 37°C, 5% CO_2, 90% RH for 1 hr then subjected to 3 min of heat in 96-well thermal cycler (Veriti – Applied Biosystems) at a temperature of 59°C. Samples were lysed, separated, and analyzed as described above. Densitometry analysis was performed using ImageQuant TL (GE Healthcare) and dose response analysis was performed using Prism (Graphpad Software).

Cytotoxicity Assay:

Cryopreserved human hepatocytes (Pool of 10 donors, Mixed gender, Cat# HUCS10P, Lot# HUP1003 Triangle Research Labs) were thawed and plated according to manufactures recommendations in a 1,536-well plate at a hepatocyte density of 2,500 cells/well in 5 μL of hepatocyte maintenance media. A 1,536 pintool (Kalypsys) was used to transfer 23 nL of compound in DMSO to the 1,536-well assay plates. After 72 hr incubation at 37°C, 2.5 μL of CellTiter-Glo (Promega) was dispensed into each well using a BiorapTR. Plates were incubated at room temperature for 10 min, transferred to a ViewLux (PerkinElmer) and the luminescence was recorded using an exposure time of 1 second.

THP1 monocytic differentiation assay:

Human THP1 leukemia cells engineered to express either wild type IDH1 or mutant IDH1-R132H under a doxycycline-inducible promoter have been previously described (7). Cells were grown in RPMI 1640 with 2 mM glutamine (Thermo Scientific) and supplemented with 10% tetracycline-free FBS (Hyclone) and 100 units/mL Penicillin, 100 ug/mL Streptomycin (Thermo Scientific) at 37°C, 5% CO_2, 90% RH. To induce transgene expression, cells were treated with doxycycline (2 µg/mL final). After 4 days of doxycycline induction, cells were passaged into a final volume of 10 mL media at a density of 1.5 x 10^5 and pre-treated with either vehicle DMSO or the indicated mIDH1 inhibitor at a final concentration of 0.5 µM for 4 additional days. Monocyte differentiation was induced by the addition of phorbol-12-mysistate-13-acetate (PMA) following a previously described protocol (8) with some modifications. Briefly, pre-treated cells were seeded into black, clear bottom 96-well plates (PerkinElmer) at a density of 1 x 10^4 cells/ 50 µl/well. An equal volume of growth media containing doxycycline (2 µg/mL final) and either DMSO or PMA (50 nM final) was added to each well and plates were incubated for 5-7 days at 37°C to allow cell differentiation and attachment. The differentiation process was induced in the continuous presence of the corresponding mIDH1 inhibitor (0.5 µM) or mDH1inhibitor with 300 µM octyl 2-HG. Final assay DMSO concentration was kept at 0.4 %. Attached cells were washed, stained, imaged and quantified as follows. Plates were turned upside down and centrifuged at 161 rcf for 10 seconds using plate adaptors to collect media containing unattached cells. A solution of 1:2000 Hoechst 33342 (Thermo Scientific) in PBS was added to each well and plates were incubated at 37°C for 15-30 min. Staining solution was removed as above and

fresh PBS was added to each well. Images were captured on the IN Cell 2200 widefield automated microscope (GE Healthcare) using a 10x 0.45 NA Plan Apo objective lens with standard Hoechst (50 msec and 100 msec exposures, 4 fields per well). Images were analyzed using IN Cell Investigator v1.6.2 analysis software's canned Multi-Target Analysis algorithm (GE Healthcare). The number of cells/well was determined as the number of Hoechst-stained nuclei (identified using Top hat segmentation with a minimum area of 75 μm^2 and sensitivity of 93). Cell viability of THP1-WT and THP1-R132H cells after 4 days doxycycline (2 μg/mL final) were determined on day 0 and day 7 post treatment of either DMSO or PMA (50 nM final) using CellTiter-Fluor™ (Promega) under identical conditions as described above except without a plate spin and staining.

Histone methylation assay:

Human THP-1 leukemia cells were grown as previously described. To induce transgene expression, cells were treated with doxycycline (2 μg/mL final) for 4 days and then pre-treated with either vehicle DMSO or the indicated mIDH1 inhibitor at a final concentration of 0.5 μM for 4 additional days. Cells ($6x10^6$) were then collected, pelleted, and snap frozen. Total histone extracts were prepared using a Histone Extraction Kit (Abcam #113476) and samples were quantified using a DC Protein Assay (Biorad). Extracts (7 μg) were separated on 4-12% Bis-Tris NuPAGE gels (Thermo Fisher) and transferred to nitrocellulose membranes using an iBlot transfer system (Thermo Fisher). Primary antibodies were diluted in TBST (50 mM Tris HCl (pH 7.5), 150 mM NaCl, 0.1 % Tween 20) + 5 % BSA and incubated

overnight at 4°C: mouse anti-H3K4me3 (Abcam #1012; 1:500), rabbit anti-H3K9me3 (Cell Signaling #13969; 1:1000) and rabbit anti-total H3 (Cell Signaling #4499; 1:2000). Membranes were washed 3 x 10 min with TBST and incubated with HRP-conjugated secondary antibodies (1:5000 in TBST + 1% BSA) for 1 hr at room temperature. SuperSignal West Dura chemiluminescence substrate (Thermo Fisher) was added and immunoblots were imaged using a BioRad ChemiDoc Imager. Densitometric analysis was performed using ImageJ.

In Vitro Physicochemical and Drug Metabolism Studies:

High-Throughput PAMPA assay

The stirring double-sink PAMPA method patented by pION Inc. (Billerica, MA) was employed to determine the permeability of compounds via PAMPA passive diffusion. The PAMPA lipid membrane consisted of an artificial membrane of a proprietary lipid mixture and dodecane (Pion Inc.), optimized to predict gastrointestinal tract (GIT) passive diffusion permeability, was immobilized on a plastic matrix of a 96 well "donor" filter plate placed above a 96 well "acceptor" plate. A pH 7.4 solution was used in both donor and acceptor wells. The test articles, stocked in 10 mM DMSO solutions, were diluted to 0.05 mM in aqueous buffer (pH 7.4) , and the concentration of DMSO was 0.5% in the final solution. During the 30-min permeation period at room temperature, the test samples in the donor compartment were stirred using the Gutbox technology (Pion Inc.) to reduce the unstirred water layer. The test article concentrations in the donor and acceptor compartments were measured using an UV plate reader (Nano Quant, Infinite 200

PRO, Tecan Inc., Männedorf, Switzerland). Permeability calculations were performed using Pion Inc. software and were expressed in units of 10^{-6} cm/s.

High-Throughput kinetic solubility test assay

Pion's patented μSOL assay for kinetic solubility determination was used. In this assay, the classical saturation shake-flask solubility method was adapted to a 96-well microtiter plate format and a co-solvent method with n-propanol as the reference compound was utilized. Test compounds were prepared in 10 mM DMSO solutions (45 μL), and diluted with the co-solvent to a final drug concentration of 150 μM in the aqueous solution (pH 7.4). Samples are incubated at room temperature for 6 hrs to achieve equilibrium. The samples were then filtered to remove any precipitate formed. The concentration of the compound in the filtrate was measured by UV absorbance. The reference drug concentration of 17 μM was used for quantitation of unknown drug concentration in filtrate. Spectroscopically pure 1-Propanol was used as a cosolvent to suppress precipitation in the reference solutions. The kinetic solubility (μg/mL) was calculated with using the μSOL Evolution software.

Microsomal metabolic stability assay

Multiple time-point metabolic stability assay (multiple species) was run in triplicates using the substrate depletion method. Incubation and liquid handling were carried out using Tecan EVO robotic system equipped with EVOware software. Briefly, each reaction mixture (110 μL) consisted of a test article (1 μM), 0.5mg/mL of microsomes, and NADPH regenerating system (1 μM) in phosphate buffer at pH 7.4. Samples were incubated in 384-well plates at 37°C for 0, 5, 10, 15, 30 and 60

min. Reaction was stopped by adding cold acetonitrile (ACN) with internal standard (albendazole). All plates were centrifuged at 3000 rpm for 20 min at 4°C, and the supernatants were analyzed using previously described method (9).

Human plasma protein binding assay

Assay was performed by Cyprotex. This study was performed under non-GLP conditions. All work was performed with appropriate local health regulations and ethical approval. Briefly, IDH inhibitors were added to human plasma at a concentration of 5 μM and the mixture was added to a RED device (Rapid Equilibrium Dialysis, Pierce) against PBS for 4 hrs at 37°C. After incubation aliquots from both sides were collected and normalized. Proteins were removed through a methanol extraction prior to analysis by LC-MS/MS. The extent of binding is reported as fraction unbound.

Caco-2 permeability assay

Assay was performed by Cyprotex. Briefly, Caco-2 cells were cultured, plated in a Millipore Millicell 96 well plate, and allowed to differentiate for 3 weeks. Cell monolayers were analyzed using Lucifer yellow. IDH inhibitors were added to either the apical or basolateral side at 10 μM and incubated for 2 hrs. After incubation both sides were analyzed by LC-MS/MS. Data are expressed as permeability (P_{app}) and Efflux ratio.

Bidirectional MDCK-MDR1 permeability assay

Assay was performed by Cyprotex. Briefly, MDCK-MDR1 cells were cultured, plated in a Millipore Millicell 96 well plate, and allowed to differentiate for 4 weeks. Cell monolayers were analyzed using Lucifer yellow. Control and AG-120 were added to

either the apical or basolateral side at 10 µM and incubated for 2 hrs. For MDR1 inhibition studies, chemical inhibitor (100 µM of Verapamil or 1 µM of Tariquidar) were added to both apical and basolateral sides during a 30 min pre-incubation as well as the 2 hr transport assay. After incubation both sides were analyzed by LC-MS/MS. Data are expressed as permeability (P_{app}) and Efflux ratio.

Determination of hERG inhibitory activity:

We implemented a previously described homogeneous 1,536-well FluxOR-based thallium flux assay with some modifications (10). Briefly, U2OS cells (ATCC) were grown to ~80% confluence, harvested and transduced with a hERG-BacMam virus (Montana Molecular) at a MOI of 1:100 for 4 hrs at room temperature protected from light. Cells were subsequently plated into black, clear bottom, TC-treated 1,536- well plates (Greiner BioOne) at a density of 1,000 cells/well/3 µL and plates were incubated ~16 hr at 37°C, 5% CO_2, 85% RH. hERG activity was determined using the FluxOR potassium ion channel assay (Themo Scientific) with some modifications. Two µl of loading buffer (HBSS without Ca^{+2}/Mg^{+2}, 20 mM HEPES, 10 mM Red 40 dye quencher, 0.77 mg/ µL probenecid and 1X FluoXOR dye) was dispensed into each well and plates were incubated at room temperature for 40 min. Assay was read using a FDSS 7000 kinetic plate reader (Hamamatsu) equipped with a pintool and tip head in 1 second intervals for ~320 seconds using a standard Fluo-4 480-nm excitation and 530-nm emission filter set. Compounds were tested at a concentration range of 45.8 µM - 1.4 nM. Plates were pinned and read for ~260 seconds at which point, hERG channels were stimulated by the addition of 1 µl of

stimulation buffer (prepared as per manufacturer's instructions) and plates were read for the remaining ~60 seconds. Data was normalized to DMSO and haloperidol controls and analyzed as described (10).

Statistical Methods:

Correlations were calculated in Prism 7 using linear regression analysis and Person's correlation coefficients. Dose response curves and parameters were calculated using the following equation –

Y=Bottom + (Top-Bottom)/(1+10^((LogEC50-X)*HillSlope))

Heat maps were generated using the derived EC50 values from the equation above which were then inputted into TIBCO's Spotfire 6.0. Hierarchical clustering was performed using the UPGMA clustering method with Euclidean distance measures. Statistical methods used for the THP1 differentiation experiments in figure 6 used two-way ANOVA with Tukey's multiple comparisons test. All comparisons values can be found in supplemental table 3.

References

1. Deng G, Shen J, Yin M, McManus J, Mathieu M, Gee P*, et al.* Selective inhibition of mutant isocitrate dehydrogenase 1 (IDH1) via disruption of a metal binding network by an allosteric small molecule. J Biol Chem **2015**;290:762-74
2. Davis MI, Gross S, Shen M, Straley KS, Pragani R, Lea WA*, et al.* Biochemical, cellular, and biophysical characterization of a potent inhibitor of mutant isocitrate dehydrogenase IDH1. J Biol Chem **2014**;289:13717-25
3. Rohle D, Popovici-Muller J, Palaskas N, Turcan S, Grommes C, Campos C*, et al.* An inhibitor of mutant IDH1 delays growth and promotes differentiation of glioma cells. Science **2013**;340:626-30
4. Dang L, White DW, Gross S, Bennett BD, Bittinger MA, Driggers EM*, et al.* Cancer-associated IDH1 mutations produce 2-hydroxyglutarate. Nature **2009**;462:739-44
5. Balss J, Pusch S, Beck AC, Herold-Mende C, Kramer A, Thiede C*, et al.* Enzymatic assay for quantitative analysis of (D)-2-hydroxyglutarate. Acta Neuropathol **2012**;124:883-91
6. Jafari R, Almqvist H, Axelsson H, Ignatushchenko M, Lundback T, Nordlund P*, et al.* The cellular thermal shift assay for evaluating drug target interactions in cells. Nat Protoc **2014**;9:2100-22
7. Chan SM, Thomas D, Corces-Zimmerman MR, Xavy S, Rastogi S, Hong WJ*, et al.* Isocitrate dehydrogenase 1 and 2 mutations induce BCL-2 dependence in acute myeloid leukemia. Nat Med **2015**;21:178-84
8. Uchide N, Toyoda H. A new simple multi-well plate-based assay for monocyte differentiation using human monocytic leukemia THP-1 cells. J Immunol Methods **2007**;328:215-9
9. Shah P, Kerns E, Nguyen DT, Obach RS, Wang AQ, Zakharov A*, et al.* An Automated High-Throughput Metabolic Stability Assay Using an Integrated High-Resolution Accurate Mass Method and Automated Data Analysis Software. Drug Metab Dispos **2016**;44:1653-61
10. Titus SA, Beacham D, Shahane SA, Southall N, Xia M, Huang R*, et al.* A new homogeneous high-throughput screening assay for profiling compound activity on the human ether-a-go-go-related gene channel. Anal Biochem **2009**;394:30-8

Supplemental Figure 1 – Cell based Assays. A, Dose-response analysis of mIDH2 inhibitor effects on R172Q mIDH2 enzyme. Dose-response analysis of mIDH1 inhibitor effects on cellular 2-HG production in (B) THP1 (R132H) and (C) SNU1079 (R132C) cell lines. D, Standard curve of 2-HG generated by the D2HGDH assay with a linear regression analysis (R^2 = 0.94, P value = <0.0001). Dotted red line represents average concentrations of 2-HG measured in media from the HT1080 cell line after 48 hrs (n=2). Dot plot of (E) H3K9me3 and (F) H3K4me3 densitometry values normalized to H3 levels and WT levels.

Supplemental Figure 2 – Differentiation assay images.
Representative images from a quadrant of a well in a 384 well plate captured with an IN Cell 2200 wide field automated microscope (GE Healthcare) using a 10x 0.45 NA Plan Apo objective lens with standard Hoechst (50 msec and 100 msec exposures). Images were captured following the removal of media using an upside-down centrifugation step.

Supplemental Figure 3 – THP1 viability, MDR1 permeability, hepatocyte toxicity, and hERG assays. THP1 cell viability assay at (A) 0 days and (B) 7 days after DMSO or PMA treatment. C, Bidirectional MDCK-MDR1 permeability assay using Loperamide (Pgp substrate), Loperamide + Tarquidar (Pgp inhibitor), Loperamide + Verapamil (Pgp inhibitor), AG-120 (clinical mIDH1 inhibitor), AG-120 + Tarquidar (Pgp inhibitor), and AG-120 + Verapamil (Pgp inhibitor). D, Bidirectional MDCK-MDR1 permeability assay results graphed using efflux ratio (P_{app} (B→A) / P_{app} (A→B)) values. E, Dose response analysis of mIDH1 inhibitor effects on human hepatocyte cytotoxicity after a 72 hour incubation. F, Dose response analysis of mIDH1 inhibitor effects on hERG channel inhibition.

Supplemental Figure 1

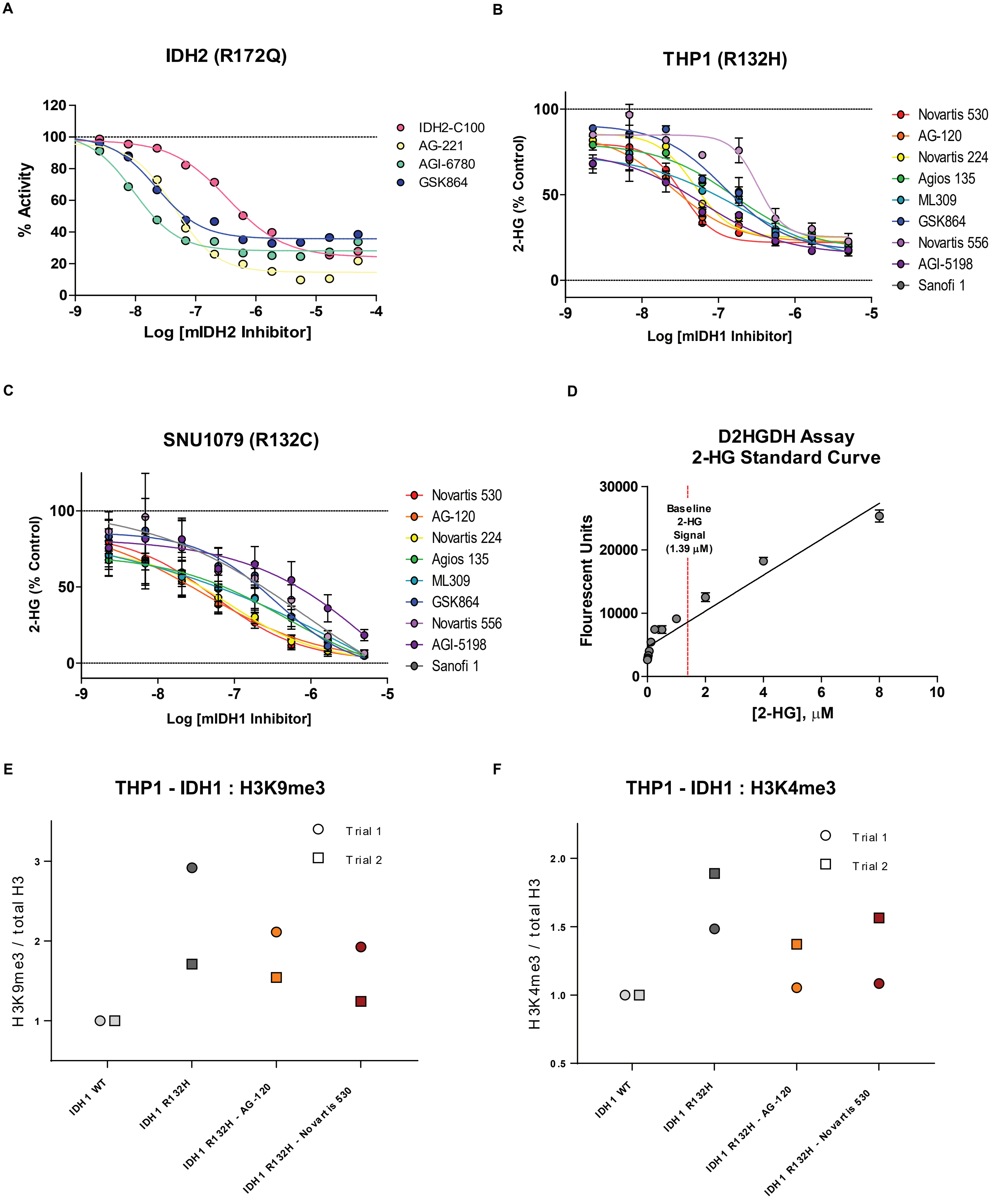

Supplemental Figure 2

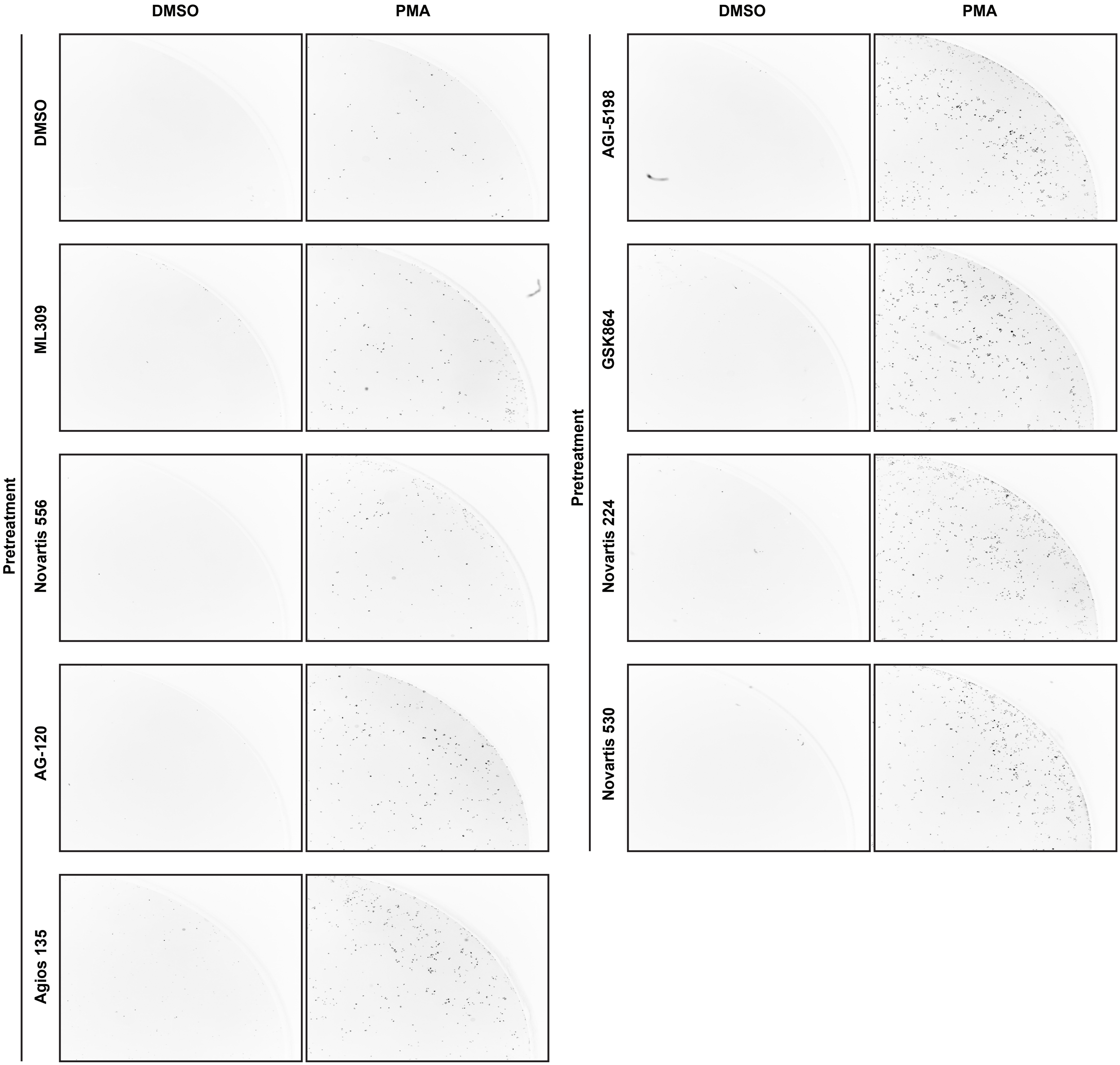

Supplemental Figure 3

A

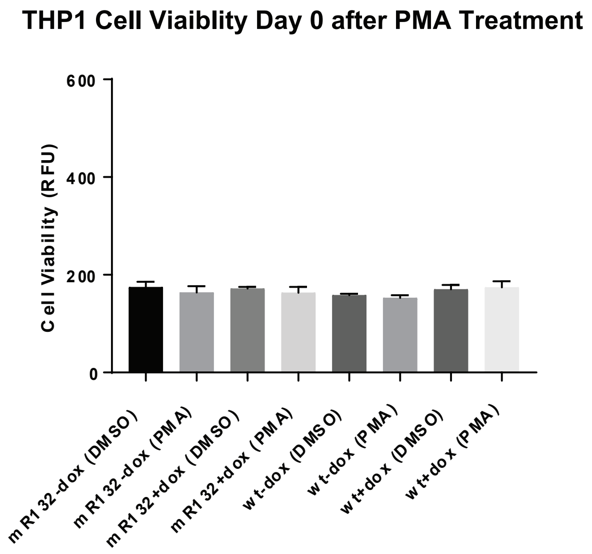

B

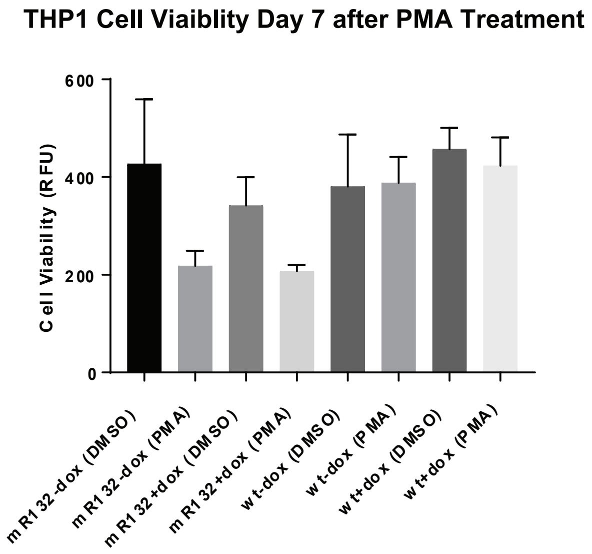

A

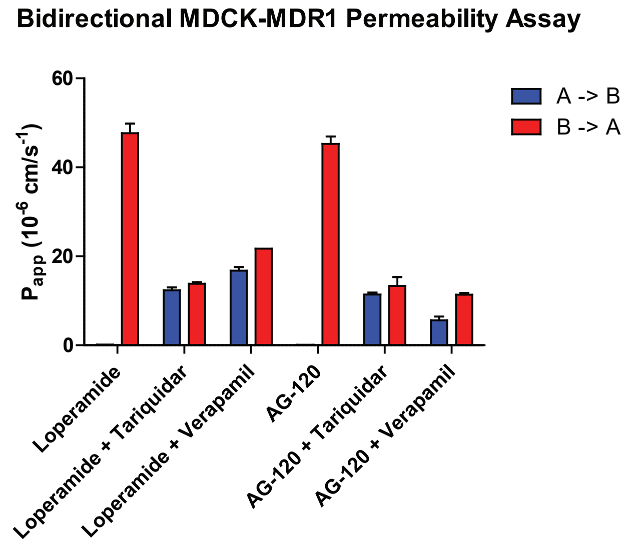

B

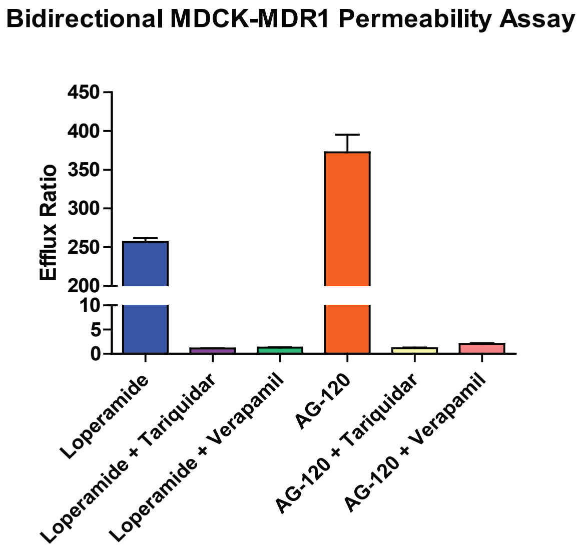

C

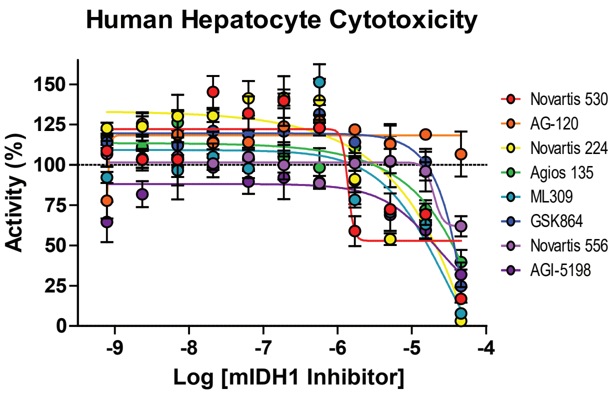

D

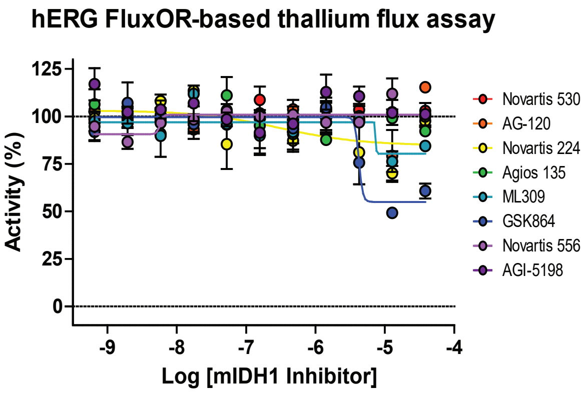

Supplement: Supplementary file 2 — Supplementary info [file 41598_2017_12630_MOESM2_ESM.pdf]
